# Co-occurrence of anaerobic bacteria in colorectal carcinomas

**DOI:** 10.1186/2049-2618-1-16

**Published:** 2013-05-15

**Authors:** René L Warren, Douglas J Freeman, Stephen Pleasance, Peter Watson, Richard A Moore, Kyla Cochrane, Emma Allen-Vercoe, Robert A Holt

**Affiliations:** 1 BC Cancer Agency, Michael Smith Genome Sciences Centre, Vancouver, BC V5Z 1L3, Canada; 2 BC Cancer Agency, Deeley Research Centre, Victoria, BC V8R 6V5, Canada; 3 Faculty of Health Sciences, Simon Fraser University, Burnaby, BC V5A 1S6, Canada; 4 Department of Molecular and Cellular Biology, University of Guelph, Guelph, ON N1G 2W1, Canada; 5 Department of Molecular Biology and Biochemistry, Simon Fraser University, Burnaby, BC V5A 1S6, Canada; 6 Department of Medical Genetics, University of British Columbia, Vancouver, BC V6T 1Z4, Canada

**Keywords:** Anaerobe, Cancer, Emerging pathogens, Host-microbe interaction, Microbial genomics, Microbiome

## Abstract

**Background:**

Numerous cancers have been linked to microorganisms. Given that colorectal cancer is a leading cause of cancer deaths and the colon is continuously exposed to a high diversity of microbes, the relationship between gut mucosal microbiome and colorectal cancer needs to be explored. Metagenomic studies have shown an association between *Fusobacterium* species and colorectal carcinoma. Here, we have extended these studies with deeper sequencing of a much larger number (n = 130) of colorectal carcinoma and matched normal control tissues. We analyzed these data using co-occurrence networks in order to identify microbe-microbe and host-microbe associations specific to tumors.

**Results:**

We confirmed tumor over-representation of *Fusobacterium* species and observed significant co-occurrence within individual tumors of *Fusobacterium*, *Leptotrichia* and *Campylobacter* species. This polymicrobial signature was associated with over-expression of numerous host genes, including the gene encoding the pro-inflammatory chemokine Interleukin-8. The tumor-associated bacteria we have identified are all Gram-negative anaerobes, recognized previously as constituents of the oral microbiome, which are capable of causing infection. We isolated a novel strain of *Campylobacter showae* from a colorectal tumor specimen. This strain is substantially diverged from a previously sequenced oral *Campylobacter showae* isolate, carries potential virulence genes, and aggregates with a previously isolated tumor strain of *Fusobacterium nucleatum*.

**Conclusions:**

A polymicrobial signature of Gram-negative anaerobic bacteria is associated with colorectal carcinoma tissue.

## Background

A substantial portion of the cancer burden worldwide is attributable to microbial pathogens [1]. Certain tumor viruses, such as human papilloma virus, have the capability of initiating tumorigenesis and are well established as etiological agents. Although it is generally the case that only a minority of infected individuals progress to cancer, in principle, an overall reduction in the incidence of cancer can be achieved by reducing the incidence of infection. Likewise, a link between *H. pylori* infection and gastric carcinoma has been well established by more than two decades of intensive research and, although the precise mechanism of tumor induction remains unknown, it is possible to reduce the risk of gastric cancer by diagnosis and treatment of *H. pylori*-induced gastritis. Hence, there are strong precedents for targeting oncogenic infectious agents for the purpose of cancer control, and motivation to explore the possibility of infectious agent involvement in other cancers. Even in the absence of any etiological role, a microbe or microbial signature with tumor specificity has potential utility for diagnosis and risk assessment.

Metagenomic analysis, whereby the presence of a microbe in a sample is inferred from the presence of its sequence signature, has become a sensitive method for identifying novel tumor-associated microbes in a culture-independent manner [2,3]. Previously, we used this method to evaluate 11 subjects with colorectal carcinoma and identified substantial over-representation of sequences mapping to *Fusobacterium nucleatum* (*F. nucleatum*) in colorectal carcinoma (CRC) tissue compared to adjacent non-tumor gut mucosal control tissue from the same subjects [4]. This observation was verified in additional CRC subjects using a quantitative PCR assay, which targeted a *F. nucleatum* genome locus. We observed significant tumor over-abundance in the cohort as a whole, and extreme over-abundance in approximately 25% of subjects. An independent study of CRC, published at the same time as ours by another team of investigators, used a nearly identical study design and obtained nearly identical results, with a broad, significant tumor over-representation of *Fusobacterium* spp. and extreme tumor over-representation in a subset of subjects [5]. Together, these reports [4,5] show an association between *Fusobacterium* spp. and CRC, and highlight the possibility of a CRC subtype where *Fusobacterium* spp. may be particularly pertinent. An association between *Fusobacterium* spp. abundance and metastasis was also observed in the above studies [4,5], and a new study has found an association between *Fusobacterium* spp. and colorectal adenomas [6].

*F. nucleatum* is a known invasive [7] and pro-inflammatory agent [8,9] that can cause acute and chronic oral [10] and gastrointestinal infections [11]. We have isolated *Fusobacterium* spp. from CRC tissue and from intestinal biopsy samples taken both from healthy individuals and Crohn’s disease patients [12,13]. The reason why *Fusobacterium* spp. may in some circumstances be pathogenic and at other times apparently benign commensal organisms is not understood. These organisms are highly adherent [14] and will attach to host epithelial cells and certain other bacterial species, but generally not to other fusobacteria. Thus, in some circumstances, the pathogenicity of *Fusobacterium* spp. is thought to be related to their ability to act as a vector, whereby they facilitate host tissue infection by co-adherent bacteria [15].

Our initial metatranscriptomic analysis of CRC (n = 11) had limited power to detect rarer microbes represented differentially in tumor and matched normal control tissue, or to place the observed differential representation in the context of the larger diversity of the mucosal microbiome. Here, we report a metagenomic analysis of a much larger independent cohort of CRC patients (n = 65). We confirm significant tumor over-representation of *Fusobacterium* spp*.* sequences and we also observe over-representation in tumor of sequences from additional, less abundant bacteria, including members of the genera *Campylobacter*, *Leptotrichia* and *Selenomonas*. There is significant co-occurrence of these genera with *Fusobacterium* and together they define a metagenomic signature of CRC.

## Methods

### Clinical specimens

For all cases, surgical samples were obtained with informed consent by the BC Cancer Agency Tumor Tissue Repository (BCCA-TTR) [16] which operates as a dedicated biobank with approval from the University of British Columbia-British Columbia Cancer Agency Research Ethics Board (BCCA REB). The BCCA-TTR platform are governed by Standard Operating Procedures (SOPs) that meet or exceed the recommendations of international best practice guidelines for repositories (NCI Office of Biorepositories and Biospecimen Research. NCI Best Practices for Biospecimen Resources, 2007). Specimens are handled with very close attention to maintaining integrity and isolation. Overall average collection time (time from removal from surgical field to cryopreservation in liquid nitrogen) for all CRC cases in the BCCA-TTR is 31 min. For this study biospecimens were held briefly at −20°C during frozen sectioning, using 100% ethanol to clean the blade between all samples. For each of the 65 subjects in our study, one tumor section and one matched control specimen were analyzed, totaling 130 samples.

### Metatranscriptomic analysis

Library construction and Illumina sequencing were performed as previously described [2,4]. Briefly, frozen tissue was homogenized in 600 μL RLT buffer (Qiagen) and passed five times through a syringe fitted with a 20 G needle. RNA was purified using the RNeasy Plus Mini Kit (Qiagen) following the manufacturer's instructions. Genomic DNA contamination was reduced using an on-column DNase I treatment according to the kit protocol. RNA quality and concentration was assessed using Agilent Bioanalyzer 2000 RNA Nanochips. Ribosomal RNAs were depleted from 1 mg of total RNA using the manufacturer’s protocol for the RiboMinus Eukaryote Kit for RNA-Seq (Invitrogen). Depletion was assessed using Agilent Bioanalyzer 2000 RNA Nanochips. Samples were found to have approximately 30% residual ribosomal RNA content (Additional file 1: Table S1) and were processed as described previously [17,18] for the construction of Illumina libraries, with the following modifications: each paired-end library was PCR amplified for 15 cycles using the standard Illumina PE1 PCR primer plus a modified PE2 primer including a unique six base insertion as an index sequence. Libraries prepared using indexed primers were then combined in two pools of 60 and 70 samples, respectively, with each pool containing both tumor and control libraries. Libraries were gel purified to remove residual adapter dimers and then sequenced on the Illumina HiSeq^tm^ 2000 platform. Four paired-end 100 nt sequence lanes were run per multiplexed library for a total of 8 lanes, yielding 564.4 million raw read pairs. Reads were aligned on a per-lane basis against a sequence database of human rRNA [19] using short read aligner BWA [20] (v0.5.9 -o 1000). Aligned reads, reads having stretches of homopolymeric DNA bases and poor quality bases were discarded. The remaining paired reads (334.5 million or 59.3% of total sequence) were aligned using BWA with the same parameters against 155,209 transcript sequences [19], after which unmapped paired reads subtracted and re-aligned against the NCBI reference human genome sequence [21]. A total of 390 million read pairs (69.1%) aligned to human sequence databases and were subtracted (Additional file 1: Table S1). The remaining unmapped read pairs were aligned against a sequence database of bacterial and viral RefSeq genome sequences from Genbank [22] and reference bacterial genome sequence from HMP [23] using Novoalign (http://www.novocraft.com; v2.7.9 -o SAM -r A -R 0), tracking unique alignments within genera and species. We chose to run Novoalign rather than BWA for the last step of the microbe profiling pipeline because it is a more permissive aligner that reports all high scoring alignments for a given read pair, which facilitates the unambiguous assignments of read pairs. Paired sequence reads that mapped both as top hits and as unique hits (*i.e.*, to a single species or genus) were tallied and organized by organism at the genus or species level using the binomial nomenclature (Additional file 2: Table S2 and Additional file 3: Table S3). All pairs that satisfied these conditions and aligned within Novoalign’s allowable mismatches (≤16 per pair of reads) were tracked. Thus, all 100 nt read pairs aligning within 92% sequence identity or better were tallied. A total of 4.7 million read pairs (0.8% of total raw sequenced pairs) aligned to microbial genome sequences using the approach described here. Read pair counts were then normalized to account for the variation in the amount of raw sequence data generated per sample. Briefly, the average number of raw reads for all samples was determined. The number of raw reads from each sample, as a proportion of the average read count, was also determined and used as a correction factor. For each sample, the number of reads mapping to each microbe was divided by the correction factor specific to that sample to obtain the normalized count. Samples were further arranged by biospecimen type (*i.e.*, normal *vs.* tumor) and the significance of differentially abundant microbes, as inferred from differentially abundant and uniquely mapped read pairs, using the R function of Metastats [24].

Microbe co-occurrence was investigated by selecting any two genera and calculating a Pearson correlation R between their sequence pair count. Read counts for the two genera were then re-assigned to sample identifiers randomly, 1,000 times, and the Pearson correlation re-assessed and the correlation R between two genera was assigned a bootstrap *P* value equal to the proportion of randomizations that resulted in an R value equal or greater to the initial R value calculated from the non-randomized data.

Correlations among genera were plotted as a network using Cytoscape v2.8.2 [25], with the color intensity of each edge corresponding to the strength of the Pearson correlation, lighter to darker matching Pearson R = 0.5046 to R = 0.9909.

Principal component analysis was performed using the ade4 package in R [26] to determine whether tumor and normal control tissue could be distinguished, globally, by microbiome content.

### Host gene expression profiling

Read pairs aligning to Ensembl human transcripts that had been segregated during the filtering steps described above were analyzed further. The number of read pairs matching each Ensembl transcript were counted and then, because Ensembl transcripts are partially redundant due to alternative splicing, read-pair counts from transcripts were consolidated by Ensembl gene IDs, to provide a read-pair count for each gene. After normalizing, as above, for raw sequence depth, we calculated the Pearson correlation R between ratios (tumor/normal) of read-pairs mapping to each distinct human gene to ratios of read-pairs mapping to each distinct bacterial genus.

To test significance, read-pair ratios were randomly re-assigned to sample identifiers 1,000 times and the Pearson correlations re-assessed. Each correlation was assigned a bootstrap *P* value equal to the proportion of randomizations that resulted in an R value equal or greater to the initial R value calculated from the non-randomized data.

### HLA prediction

HLA class I alleles were predicted directly from the RNA-Seq data as described [27]. Briefly, HLA sequence contigs were derived from the RNA-Seq data by targeted *de novo* assembly using TASR [28] and assessed by reciprocal BLASTN [29] against reference HLA allelic sequences available in the public domain (IMGT/HLA) [30]. Top scoring allele predictions for all 65 subjects are provided in Additional file 4: Table S4. For the 14 subjects with the predicted HLA class I extended haplotype A*01 B*08 C*07 [31], the computational predictions were verified by PCR based Sanger re-sequencing as previously described [32].

#### *Campylobacter showae* CC57C sequencing, assembly and annotation

A strain of *Campylobacter showae* (*C. showae*), which we named CC57C, was cultured from a tumor surgical section using previously described methods [4]. CC57C was cultured on agar media supplemented with sodium formate (0.2% w/v) and sodium fumarate (0.3%w/v) [33], HMW genomic DNA was extracted and purified, and used to construct an Illumina whole genome shotgun sequencing library using standard methods. The library was sequenced on the Illumina MiSeq platform which generated 1.8 million paired 150 nt reads after quality filtering, providing 245.4 mean fold coverage of the approximately 2.2 Mbp genome. Read pairs were assembled with the onboard Velvet short read assembler [34], producing 300 contigs with an N50 length of 16.8 kbp. The contigs were aligned onto the closest known reference genome, *C. showae* RM3277 (NCBI Reference Sequence: NZ_ACVQ00000000.1) using cross_match (parameters -minmatch 29 -minscore 59 -masklevel 101, http://www.phrap.org) and displayed using XMatchView (http://www.bcgsc.ca/platform/bioinfo/software/xmatchview). CC57C genome annotation was inferred from BLASTX alignments between CC57C contigs and the protein database Genbank-nr [22].

#### Assessment of *F. nucleatum* CC53 / *C. showae* CC57C co-aggregation

*F. nucleatum* tumor isolate strain CC53 identified in a previous study by our group [4] was cultured in tryptic soy broth supplemented with menadione (1 μg/mL) and hemin (5 μg/mL) (TSBsupp), and *C. showae* tumor isolate strain CC57C was cultured in TSBsupp with further supplementation of sodium formate (0.2% w/v) and sodium fumarate (0.3% w/v), under strict anaerobic conditions for 2 days at 37°C.

To assess whether CC53 and CC57C were able to co-aggregate, cells were harvested by centrifugation at 10,000 rpm for 10 minutes at 4°C, washed in co-aggregation buffer (0.01 M Tris–HCl adjusted to pH 8.0, 0.001 M MgCl_2_, 0.15 M NaCl and 2% NaN_3_) ref. three times and finally re-suspended in co-aggregation buffer to a turbidity of between McFarland standard 2.0 and 3.0 (~108 CFU/mL). Aliquots (0.5 mL) of each strain were mixed and vortexed for 10 s followed by incubation at 37°C for 30 min with agitation at 110 rpm. After incubation the tubes were removed and allowed to sit undisturbed for 3 min to allow aggregates to settle. Aggregate samples were immobilized on agarose-coated slides and observed by phase microscopy.

### Preparation of cells for electron microscopy

CC57C cells were cultured in TSBsupp with further supplementation of sodium formate (0.2% w/v) and sodium fumarate (0.3% w/v) as above for two days as above. Cells were harvested by centrifugation, washed twice with distilled water, floated to Formvar-coated grids, and stained with 0.5% uranyl acetate prior to viewing with a Philips CM10 electron microscope.

## Results

### Metatranscriptomic analysis of CRC and matched control tissue reveals differentially abundant microbes

Total RNA was isolated from frozen surgical sections of CRC and matched unaffected control tissue from 65 subjects. Following ribosomal RNA depletion, multiplexed RNA-Seq libraries were constructed as previously described [2,4], and eight paired-end lanes of sequence were obtained using the Illumina HiSeq^tm^ 2000 platform. After quality filtering of the raw sequence data and removal of human sequences, microbial sequence diversity was assessed by counting read pairs having unique alignments to genomes of specific bacterial genera, using an in-house database populated with all microbial genome sequences available from GenBank [22] and the Human Microbiome Project [35]. Aligned read pairs were normalized to correct for differential yield of raw sequence data from different samples. Considering the filtered and uniquely mapped sequences of microbial origin (Table 1 and Additional file 1: Table S1 and Additional file 2: Table S2), 57 distinct genera accounted for 99% of the mapped read pairs, and this set of frequent bacteria was analyzed further. Relative microbial abundance in the tumor and normal groups was inferred from the differential abundance of uniquely mapped reads by running Metastats [24].

**Table 1:** Sequence statistics

**Sample type**			**Control**				**Tumor**	
**Number of patient samples**			**65**				**65**	
**Read pairs**	**Minimum**	**Maximum**	**Mean ± SD**	**Proportion of raw read pairs (%)**	**Minimum**	**Maximum**	**Mean ± SD**	**Proportion of raw read pairs (%)**
Raw sequenced	2,393,763	5,991,413	3,854,055 ± 731,259	100.0	1,467,161	10,121,411	4,371,501 ± 1,435,367	100.0
Quality filtered and host subtracted	747,357	2,404,589	1,259,480 ± 288,807	32.7	507,402	3,426,796	1,403,283 ± 475,582	32.0
Mapping unambiguously to microbial genomes	646	792,618	44,625 ± 103,949	1.2	445	219,314	27,191 ± 39,676	0.6
Distinct microbial genera observed			**372**				**441**	

**Figure 1:**
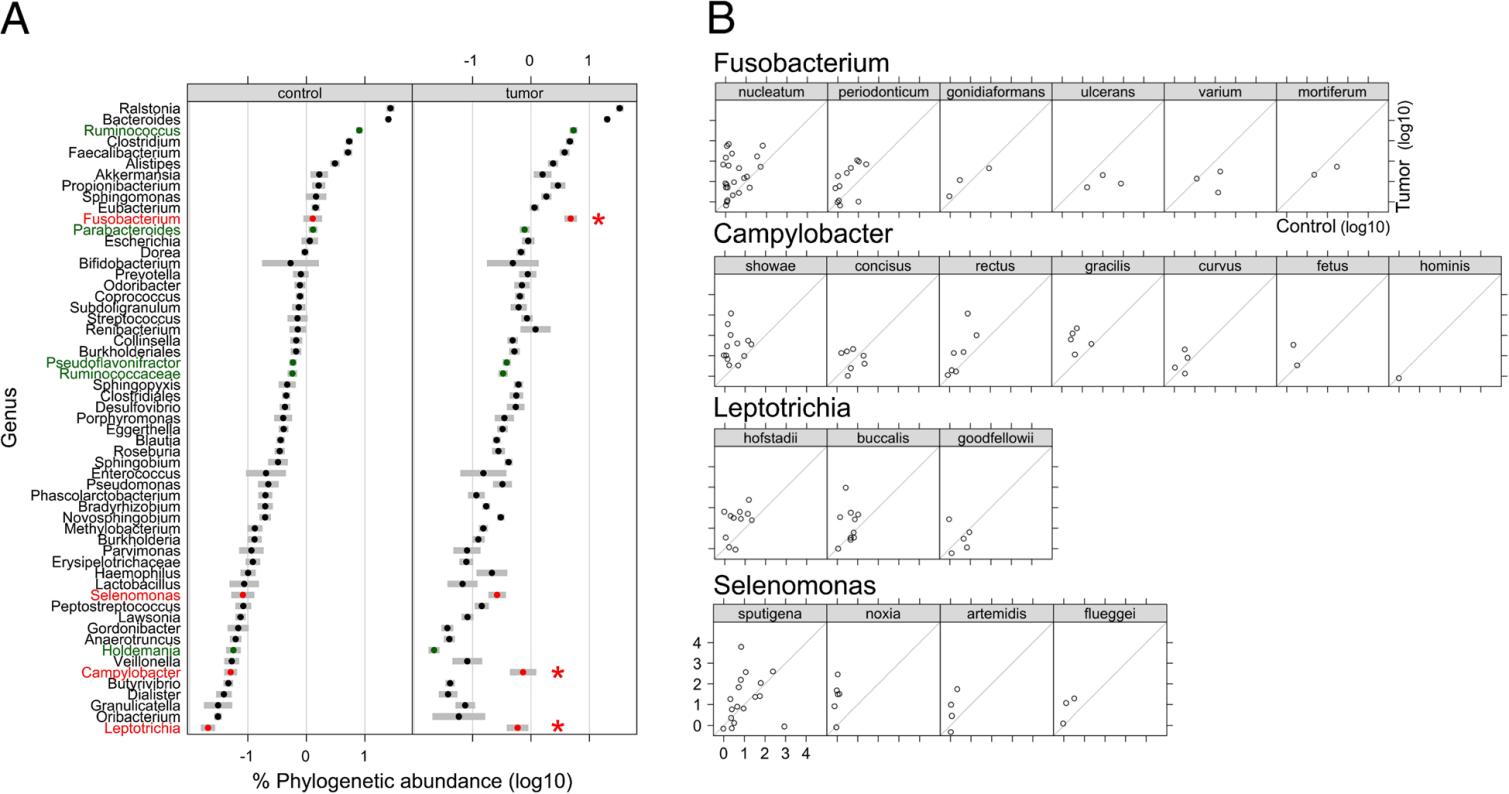
**Microbial abundance in CRC and unaffected control gut mucosa tissue measured by RNA-Seq.** (**A**) Phylogenetic abundance inferred from unique metatranscriptomics read pair mapping. Genera (n = 57) comprising, collectively, 99% of the microbial sequence data are shown. Values presented are the mean percent abundance ± SE, on a log10 scale. Genera are ordered top to bottom by decreasing read pair abundance in control tissue. The names of genera nominally over-represented in tumor (*P* <0.05) are red, and the names of genera nominally under-represented in tumor (*P* <0.05) are green. Genera indicated with an asterisk are significantly over-represented in tumor samples after multiple hypothesis testing correction (q <0.05); (**B**) Species distribution of uniquely mapped *Fusobacterium*, *Leptotrichia*, *Campylobacter* and *Selenomonas* normalized sequence pairs.

Relative abundance within the set of 57 frequent bacterial genera varied by approximately four orders of magnitude (Figure 1A; Additional file 5: Table S5). *Ralstonia* and *Bacteroides*, both of which are known constituents of the normal fecal microbiota, were of highest abundance in both tumor and control samples. Genera we found to be nominally over-represented in control samples relative to tumors (*Ruminococcus*, *Parabacteroides*, *Pseudoflavonifractor*, *Ruminococcaceae* and *Holdemania*) are also well-recognized members of the fecal microbiota [36]. *Fusobacterium* was the most abundant genus significantly over-represented in tumor samples, as previously reported [4,5]. Interestingly, however, analysis of this comprehensive metagenomic data set revealed additional genera, namely *Campylobacter* and *Leptotrichia,* which are significantly over-represented in tumor samples after accounting for multiple testing. Tumor over-representation of *Selenomonas* is only nominally significant (Additional file 5: Table S5). These additional genera have comparatively low representation in the tumor microbiome, but high tumor specificity. Principal component analysis did not distinguish tumor from normal control tissue on the basis of global microbiome content (Additional file 6: Figure S1).

To explore species-level representation of the tumor-associated genera, we counted read pairs mapping uniquely to genome accessions of member species (Figure 1B). The species showing greatest number of unique and unambiguous read pair alignments were *F. nucleatum*, *C. showae*, *L. hofstadii* and *S. sputigena*. For each of these species, there was an over-representation of mapped read pairs that were of tumor origin. Within each genus, additional species showed unique read pair matches, but the matches were generally fewer and showed less tumor specificity. We have restricted our analysis to sequence pairs that map as best hits, uniquely to the reference genomes shown because this is the most tractable approach, however, this approach cannot yield complete genome information for tumor microbes. Therefore, we cannot state with certainty that these are the precise species present. It is possible that regions of individual genomes we have not sampled would, if available, match best to reference genomes from different species. Likewise, the sequences we have obtained from tumor microbes might best match species that are not yet represented in microbial genome databases. It is expected that precision in microbial identification at the finer taxonomic levels will improve as microbial genome resources improve.

#### *Fusobacterium, Campylobacter* and *Leptotrichia* show patterns of co-occurrence in CRC

**Figure 2:**
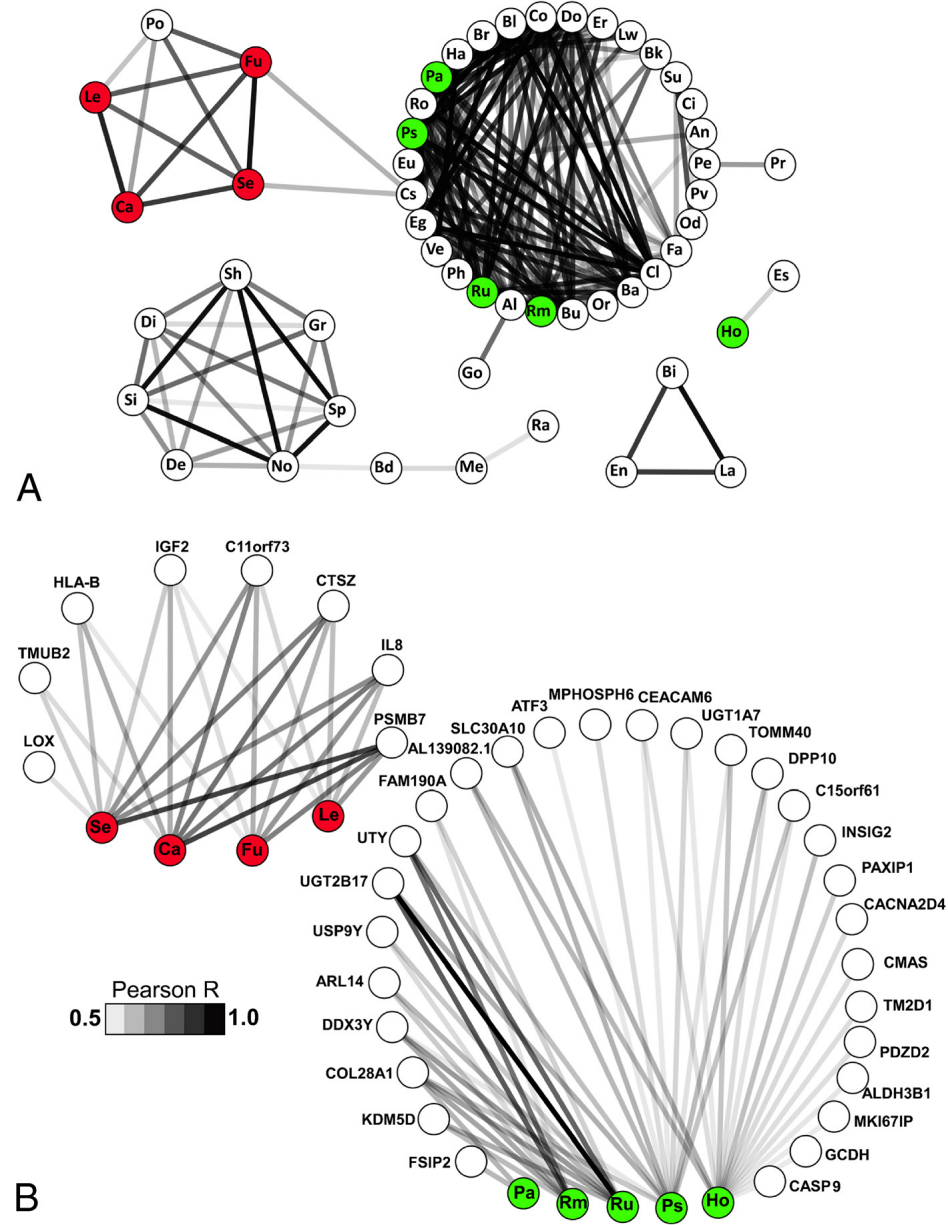
**Bacterial co-occurrence and correlation with host gene expression.** (**A**) The co-occurrence of microbes was inferred by pairwise correlation of sequence counts for all genera in Figure 1. Significance was determined by 1,000 iterations of random re-assignment of sequence read pairs to subjects, with re-calculation of Pearson correlation coefficients. Interactions of significantly differentially abundant genera are illustrated here in a network diagram, constructed using Cytoscape [25]. Pearson R values ranged from a low of 0.51 (*Holdemania-Haemophilus*) to a high of 0.97 (*Fusobacterium*-*Selenomonas*). Each prefix within a node of the network indicates a bacterial genus: Sp: Sphingobium; Sh: Sphingopyxis; Si: Sphingomonas; La: Lactobacillus; Bi: Bifidobacterium; No: Novosphingobium; Se: Selenomonas; Fu: Fusobacterium; Ro: Roseburia; Eg: Eggerthella; Bl: Blautia; Cl: Clostridium; Ve: Veillonella; Ha: Haemophilus; Co: Coprococcus; Ps: Pseudoflavonifractor; Do: Dorea; Ru: Ruminococcus; Bu: Butyrivibrio; Cs: Clostridiales; Ca: Campylobacter; Le: Leptotrichia; Pa: Parabacteroides; Ba: Bacteroides; Rm: Ruminococcaceae; Er: Erysipelotrichaceae; En: Enterococcus; Br: Burkholderia; Ph: Phascolarctobacterium; Lw: Lawsonia; Al: Alistipes; Su: Subdoligranulum; Od: Odoribacter; Po: Porphyromonas; Bk: Burkholderiales; Go: Gordonibacter; Gr: Granulicatella; Di: Dialister; Fa: Faecalibacterium; Eu: Eubacterium; De: Desulfovibrio; Pr: Parvimonas; Pe: Peptostreptococcus; An: Anaerotruncus; Ci: Collinsella; Or: Oribacterium; Ho: Holdemania; Es: Escherichia; Pv: Prevotella; Me: Methylobacterium; Bd: Bradyrhizobium; Ra: Ralstonia. (**B**) The host factor and microbe interaction was inferred by comparing the relative read pair abundance in the tumor *vs.* the control samples for each patient. The red and green nodes correspond to microbes with at least nominally significant differential abundance between tumor and control tissues.

Next, we asked if the bacteria showing mean differential abundance between the tumor and control samples showed a tendency toward co-occurrence. That is, we were interested to determine if these bacteria tended to be differentially abundant in the same samples. Using read count data, we calculated correlation coefficients for all possible pairwise combinations of genera and assessed the significance of these correlations by iterative retesting after randomization of the association between genus abundance and sample identifier. The resulting co-occurrence network is illustrated in Figure 2A and shows very distinct clustering of the tumor-enriched genera, indicating a tendency of these genera to co-occur within the same specific samples. Conversely, genera initially found to be nominally over-represented in control tissue did not specifically cluster, but rather grouped with the majority of the other non-differentially abundant genera that represent the commensal microbiota.

### Correlation between host factors and microbial abundance

Our RNA-Seq data provided only light coverage of the human transcriptome, but nonetheless this allowed the opportunity to explore patterns of host gene expression in relation to differentially abundant bacteria. We aligned the RNA-Seq data to Ensembl [19] transcripts (consolidated by Ensembl gene ID), and identified a total of 12,963 distinct expressed human genes and these varied widely in expression level (Additional file 7: Figure S2). Across all 130 tissue samples 5,384 genes were matched by an average of ten read pairs or more. Read counts for each of the nine bacterial genera showing at least nominally significant mean differential abundance between tumor and control samples (Figure 1A) were correlated with read counts for each of the 12,963 human genes. As above, significance was assessed by iterative randomization and retesting of the association between sample identifier and gene expression level. As illustrated in the network diagram presented in Figure 2B and Additional file 8: Table S6, genera over-represented in tumor tissue were associated with eight differentially expressed host genes, including known oncogenes and immune response genes. Genera under-represented in tumor tissue were associated with 28 differentially expressed host genes, including numerous housekeeping genes but no genes that have obvious, well established implications in cancer, infection, or immunity.

To further explore host immunity we predicted the HLA class I alleles for all subjects using a computational approach [27] that involved targeted assembly of RNA-Seq reads matching HLA-A, -B and -C genes [28] followed by identification of the most likely allele calls as best reciprocal BLASTN [29] matches to reference allele sequences from the IMGT/HLA database [30] (Additional file 4: Table S4). We did not observe an association between any individual HLA allele and genus with differential tumor abundance. Interestingly, however, 14 subjects were predicted to carry the extended ancestral haplotype A*01 B*08 C*07 [31] and showed a tendency toward tumor enrichment of *Fusobacterium* (tumor ratio of 126 ± 344 in A*01 B*08 C*07 subjects *versus* 30 ± 91 in other subjects), although this difference was not statistically significant (Student’s *t*-test).

### Characterization of a novel *C. showae* tumor isolate

The tissue specimens interrogated in the present study were biobank specimens and were not initially collected or stored with the intention of facilitating the subsequent culture of anaerobic bacteria. Nonetheless, given the observation from our metatranscriptomic analysis of over-representation of certain anaerobes, culture was attempted, and we were able to obtain a single *Campylobacter* spp. isolate, CC57C. Phenotypically, on agar plates, colonies of *C. showae* CC57C appear circular, small, extremely flat and opaque/translucent. By electron microscopy (Additional file 9: Figure S3) cells generally possess a single polar flagellum. This is in contrast to the description of the type strain for *C. showae*, ATCC 51146T, which has 2 to 4 unipolar flagella per cell, but similar to the phenotype for *C. rectus* and *C. concisus*, both of which are reported as having 1 polar flagella per cell [33].

**Figure 3:**
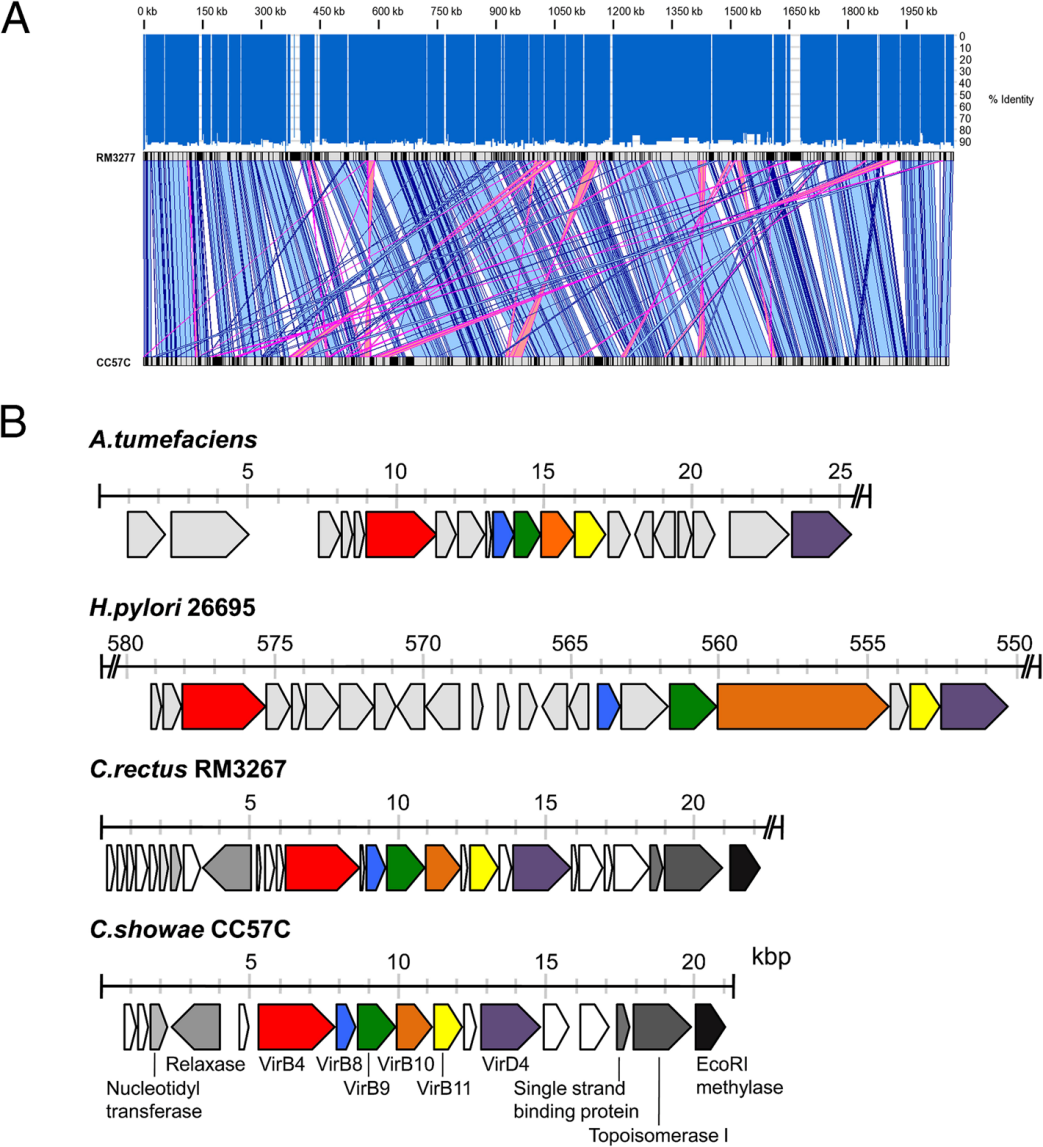
**Genome sequence analyses of *C. showae *tumor strain CC57C.** (**A**) Sequence alignments between the assemblies of the CRC tumor-associated *C. showae* CC57C genome and that of its closest known relative reference, *C. showae* RM3277. Velvet contigs from the CC57C strain were aligned to the HMP reference strain (NCBI Reference Sequence: NZ_ACVQ00000000.1) using cross_match (http://www.phrap.org), ordered and oriented based on the sequence alignments. The black and grey rectangles represent each genome sequences and indicate the absence and presence of alignments, in that order. Co-linear and inverted sequence alignment blocks are shown in blue and pink, respectively. In average, the CC57C genome assembly is 92.5% identical to that of the RM3277 strain, with 85.4% sequence coverage (represented by the dark blue and lack of bars in the histogram above the alignment, respectively). (**B**) Gene organization of the type IV secretion system (T4SS) operon in *A. tumefaciens* (plasmid pTiAB2/73 vir region AF329849), *H. pylori* strain 26695 (NC_000915.1), *C. rectus* (NZ_ACFU0100008) and *C. showae* CC57C (AOTD01000166). Components of the T4SS found in the CC57C strain are color-coded in other organisms and include VirB4 (red), VirB8 (blue), VirB9 (green), VirB10 (orange), VirB11 (yellow) and VirD4 (purple). Other annotated genes within the CC57C operon and with homologs in *C. rectus* are shown using a dark grey scale. Annotated genes in *C. rectus*, *H. pylori* and *A. tumefaciens* are shown in light grey. Hypothetical protein coding genes are depicted in white. CC57C contig AOTD01000166 with the T4SS shown in (**B**) does not have any similarity to the *C. showae* RM3277 strain and thus is not represented in the alignment shown in (**A**).

The genome of the *C. showae* CC57C isolate was sequenced using the Illumina MiSeq^tm^ platform and the resulting genome assembly showed highest DNA sequence homology with that of the HMP reference *C. showae* RM3277. However, whole-genome alignments show that these two genomes (*C. showae* CC57C and *C. showae* RM3277) are actually fairly substantially diverged, with only 92.5% average nucleotide sequence identity. Further, 15% of the *C. showae* RM3277 genome is not represented by contigs of the *C. showae* CC57C assembly and, although there is inherent difficulty in comparing draft genome assemblies, the two genomes appear to show significant local rearrangement (Figure 3A). Interestingly, there are *C. showae* CC57C sequence contigs that harbor predicted genes that have no equivalent in *C. showae* RM3277. One such contig captures VirB4/D10 operon homologs, components of a Type IV Secretion System (T4SS) specific to anaerobes, which was first described in the plant pathogen *Agrobacterium tumefaciens*[37]. T4SS are used by pathogenic anaerobic bacteria to translocate DNA and protein substrates across their membranes and into recipient cells [38]. The configuration of the Vir operon of *C. showae* CC57C (Figure 3B) shows strong similarity to that of *C. rectus* RM3267 and also similarity to that present within the *cag* pathogenicity island of *H. pylori*. In *H. pylori* this system is used to introduce the CagA oncoprotein into host stomach epithelium cells [39,40] and, although we do not observe a *cagA* equivalent in *C. showae* CC57C, the function of this secretion system in *C. showae* CC57C deserves further scrutiny, due to its potential association with virulence. The presence of Relaxase and Nucleotidyl transferase genes in the *C. showae* CC57C operon suggests a nucleic acid transport function. Annotation of the *C. showae* CC57C genome reveals additional genes of interest regarding potential pathogenicity, including genes encoding various adhesion, motility, chemotaxis and secretion functions (Additional file 10: Table S7). Numerous antibiotic resistance genes are also observed, including those coding for beta-lactamase and the multidrug resistance protein MEXB, suggesting that antibiotic treatment for this particular strain of *C. showae* may be problematic. Of note, our BLASTX-based annotation of the draft *C. showae* CC57C genome is very conservative, and we expect that many additional genes will be revealed upon further curation.

**Figure 4:**
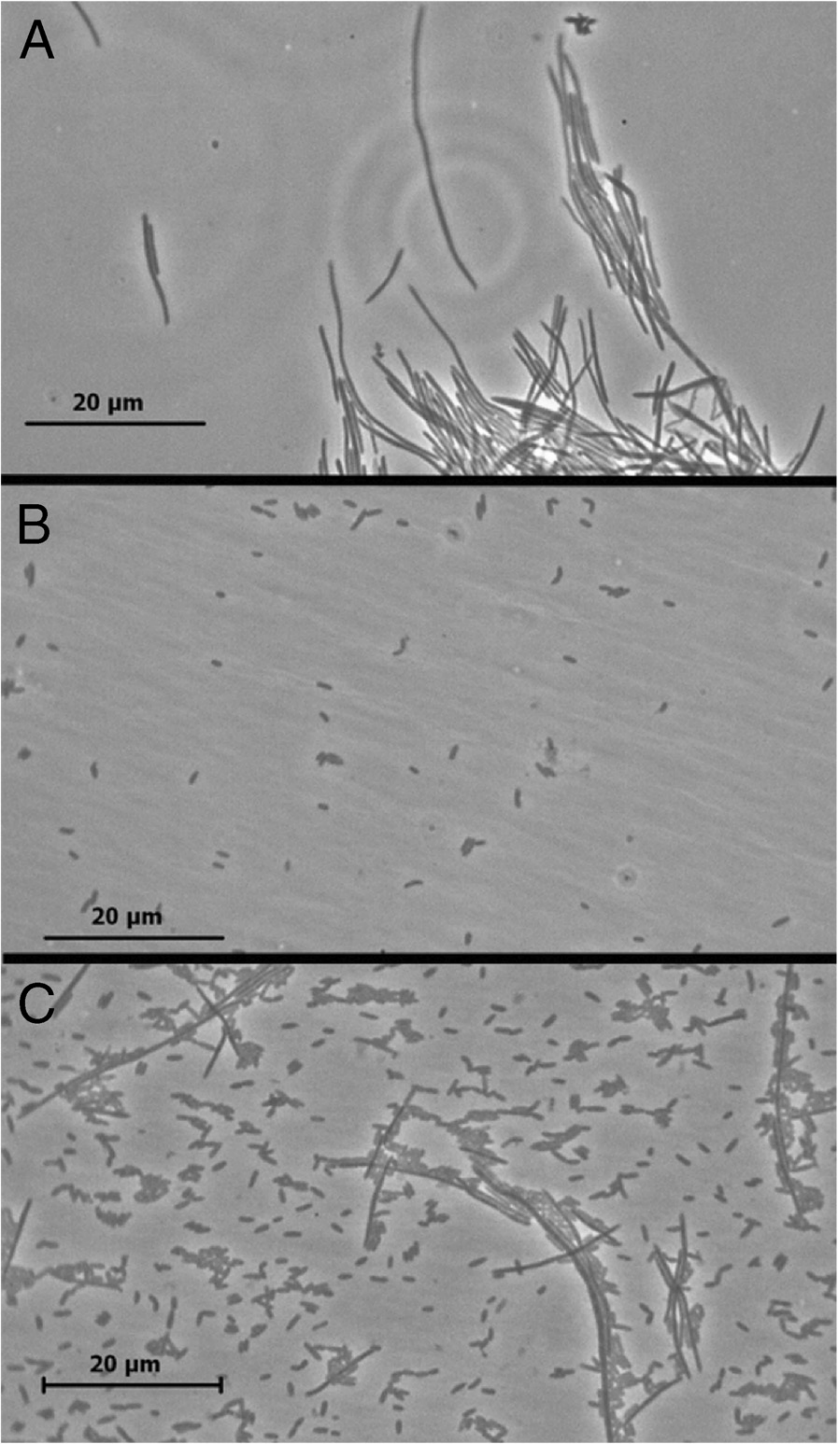
**F. nucleatum and C. showae co-aggregation.** Phase-contrast microscopy images of (**A**) F. nucleatum CC53 alone, (**B**) C. showae CC57C alone and (**C**) a mixture of CC53 and CC57C, following incubation in co-aggregation buffer (see methods). The long, thin cells of CC53 readily self-aggregate when incubated in aggregation buffer alone (panel **A**), but also show aggregative ability with the much smaller CC57C coccobacilli (panel **C**). Images were taken using a Leica DM750 microscope fitted with a 100× oil objective, using the Leica Application Suite LAS EZ Version 1.7.0 software.

The availability of *F. nucleatum* CC53 from our previously published study [4] and the availability of *C. showae* CC57C from the present study allowed us to test whether these strains interact (aggregate) with each other *in vitro* using a simple aggregation test. Using phase microscopy we found that these two strains aggregated readily with each other, indicating the presence of compatible adhesins/receptors on the bacterial cell surfaces (Figure 4).

## Discussion

Using deep metatranscriptomic sequencing and analysis we have determined that in addition to *Fusobacterium*, there are *Leptotrichia* and *Campylobacter* spp. co-enriched in CRC tissue. These bacteria are all Gram-negative anaerobes that are known commensal members of the oral microbiome, typically the subgingival plaque [33,41], and they have pathogenic potential. *Fusobacterium* and *Leptotrichia* are relatively closely-related organisms. The order *Fusobacteriales* contains three families, of which *Leptotrichiaceae* and *Fusobacteriaceae* are two. *Leptotrichia* spp. have been isolated from periodontal lesions and from a diversity of other cardiovascular, genitourinary and gastrointestinal abscesses and from systemic infections, and have been suggested to be potential emerging pathogens [42]. In our study the *Leptotrichia* spp. showing the largest number of unique read-pair alignments from our data is *L. hofstadii*, followed closely by *L. buccalis*. Previous case studies have reported *L. buccalis* bacteremia in patients with cancer [42,43], however, the cancers in question were mainly lymphomas and leukemias, and thus bacteremia was likely related to the immuno-compromised state of the patients, rather than to the malignancy directly.

The *Campylobacter* spp. showing the largest number of unique read-pair alignments from our data set was *C. showae*. This is a relatively new member of the *Campylobacter* genus, first described in 1993 [33] after isolation from subgingival plaque. *C. showae* and originally distinguished from other *Campylobacter* based on number of flagella. The presence of flagella also distinguishes *C. showae* from the other tumor-associated bacteria in our study, *Fusobacterium* and *Leptotrichia*, which are non-flagellated. Interestingly, we did not observe any unique sequence matches to *Campylobacter jejuni*, which is a well-characterized human pathogen that is a leading cause of acute, food-borne gastroenteritis [44]. Previous studies have not found epidemiological evidence for a link between *C. jejuni* infection and cancer, which is consistent with our observation of the absence of *C. jejuni* from the CRC tumor microbiome. Importantly, however, no data are available regarding the possible involvement of other *Campylobacter* species in cancer. The possibility that *Campylobacter*, *Leptotrichia*, or *Fusobacterium* spp. associated with CRC may have an etiological role in carcinogenesis remains an open and difficult question that requires further study.

The metatranscriptomic data presented here indicate that in the samples we analyzed, *Fusobacterium*, *Leptotrichia* and *Campylobacter* spp. tend to be found together. This is not unexpected given that all are anaerobic microbes known to inhabit the same niche in the oral cavity. In general, *F. nucleatum* strains are remarkable in their abilities to co-aggregate with a wide variety of bacterial species [45,46]. In line with this, we demonstrate here that, *in vitro*, aggregation of tumor isolates *C. showae* CC57C with *F. nucleatum* CC53 [4] is evident (Figure 4). Co-aggregation of *Streptococcus cristatus* with *F. nucleatum* has previously been shown to facilitate invasion of the former into cultured host cells, and to alter the host response to *F. nucleatum* invasion [47,48]. Whether co-aggregation of *F. nucleatum* and *C. showae* is relevant to disease etiology remains to be tested and the influence of this co-aggregation on bacterial virulence *in vitro* is currently being investigated by our group.

The strong association between what are typically considered oral anaerobic bacteria and colorectal carcinoma is intriguing. These species together may provide a set of parsimonious predictors with potential utility in CRC detection and risk assessment. An important question is whether these tumor bacteria are of oral origin, or if they represent distinct colonic strains or even, possibly, distinct tumor strains. Comparative analysis of large numbers of tumor isolates will be needed to address this question. Towards this goal, we describe a novel *C. showae* strain, CC57C, isolated from colorectal carcinoma tissue which shows considerable divergence from its closest known relative, *C. showae* RM3277, an oral strain. CC57C carries genes implicated previously in pathogenicity, including a virB10/D4 type IV secretion system that is present in CC57C but absent from RM3277.

An advantage of our approach of using RNA-Seq to interrogate surgical tissue for the presence of associated microbes is that it also provided the opportunity to explore possible links of the microbiota to host genetic factors. We were able to predict HLA class I alleles for most subjects by directly mining the RNA-Seq data using a new tool, HLAminer, which avoided the extra time and cost that would otherwise be required for conventional typing. Although we do not see a significant association between HLA class I type and the abundance of specific microbes, we anticipate that in future this approach will be amenable to the analysis of larger microbiome datasets that are more appropriately powered for the detection of HLA associations. We also asked if differentially abundant bacteria were associated with any differentially expressed host genes. Notably, within the small set of genes associated with tumor-enriched bacterial signatures, we observed cathepsin Z, a tumor associated protease, and interleukin-8, an inflammatory cytokine and mediator of innate immunity secreted by activated macrophages. We expect that further definition of the interaction between host cells and the anaerobic bacteria identified in this study will be an important focus of ongoing investigation.

## Conclusions

A high-throughput sequence screen of Metatranscriptome data from CRC and matched control tissues has revealed differently abundant microbial genome sequence signatures of significance in tumor samples, including those belonging to the *Fusobacterium*, *Campylobacter* and *Leptotrichia* genera. These Gram-negative anaerobes are typically considered to be oral bacteria. However, tumor isolates for *Fusobacterium* and *Campylobacter* are genetically diverged from their oral counterparts and carry potential virulence genes. Interestingly, we observe that sequence signatures from *Fusobacterium* co-occur with those from *Leptotrichia* and *Campylobacter* and that *Fusobacterium* and *Campylobacter* strains isolated from tumor tissue co-adhere in culture. A non-invasive assay to detect this polymicrobial signature of CRC may have utility in screening and risk assessment. It remains unknown whether there is any etiological link between microorganisms and CRC. In principle, any such link could provide a point of intervention.

## Availability of supporting data

The data sets supporting the results of this article are available in the NCBI Sequence Read Archive repository, http://www.ncbi.nlm.nih.gov/Traces/sra/sra.cgi under accession no. SRP010181. These include human CRC RNA-Seq as well as *C. showae* CC57C WGS reads. The whole genome shotgun project for the *C. showae* CC57C tumor isolate has been deposited at DDBJ/EMBL/GenBank under the accession AOTD00000000. The version described in this paper is the first version, AOTD01000000.

A file containing patient ID, sample ID and sequencing library names is available at http://ftp.bcgsc.ca/supplementary/CRC2012/SRP0010181_CRC_info.txt. Other data sets supporting the results of this article are included within the article and its Additional files.

## Abbreviations

CRC: Colorectal carcinoma; HLA: Human leukocyte antigen; HMP: Human microbiome project; IA: Infectious agents; T4SS: Type IV secretion system.

## Competing interests

Submitted patent CA2011/001108.

## Authors’ contributions

RAH conceived and designed the study; PW arranged the sample collection and preparation; DJF extracted RNA and constructed the sequencing libraries; RAM contributed to conception of the research project and coordinated the sequencing; KC cultured CC57C, extracted DNA for genome sequencing, and carried out aggregation assays and microscopy. SP extracted gDNA, constructed and sequenced the CC57C genome library. EAV contributed to data interpretation. RLW and RAH analyzed the data and made the figures; RLW and RAH prepared the draft manuscript. All authors discussed the results and contributed to the preparation of the final manuscript. All authors read and approved the final manuscript.

## Supplementary Material

Additional file 1: Table S1 NGS sequence read alignment summary. Click here for file

Additional file 2: Table S2 Normalized NGS sequence read pairs aligning as best hits to a unique genus. Click here for file

Additional file 3: Table S3 Normalized NGS sequence read pairs aligning as best hits to a unique genus. Click here for file

Additional file 4: Table S4 HLA allele assignments. Click here for file

Additional file 5: Table S5 Differentially represented genera from top 99% microbial abundance. Sequence reads normalized for depth of sequencing were analyzed using statistical methods in the Metastats package [24], which were designed for comparing clinical metagenomic samples from two treatment populations on the basis of read count data. Rare microbes (collectively <1% of sequence data) were excluded from analysis. Genera highlighted in **RED** are nominally over-represented in tumor tissue relative to matched normal control tissue (*P* <0.05), and genera highlighted in **GREEN** are nominally under-represented in tumor tissue (*P* <0.05). Genera shown in bold indicate significantly over-represented microbes in the tumor tissue (q <0.05). Click here for file

Additional file 6: Figure S1 Microbiome profiles of normal and tumor samples do not group separately. Principal component analysis with sample types (normal, tumor) as instrumental variables, based on the abundance of 57 genera (representing 99% of the microbe abundance) in 65 normal and 65 tumor samples. Two first components were plotted using the ade4 [26] package in R and represented 68% of the variance. CRC patients (xy points) were clustered and center of gravity (with labels normal and tumor centered in each ellipse) computed for each class. Click here for file

Additional file 7: Figure S2 Unique read pair alignment distribution. Paired read alignments were performed as described in methods, the number of raw pairs aligning unambiguously was tallied for each transcript/sample and consolidated per Ensembl gene. Click here for file

Additional file 8: Table S6 Microbe and host factor correlation. Click here for file

Additional file 9: Figure S3 Representative transmission electron microscopy image of *Campylobacter* strain CC57C, stained with 0.5% uranyl acetate. In most cases, only a single unipolar flagellum was seen associated with each cell (arrows). The lack of sharply defined edges around cells may indicate the presence of a capsule. Image taken using a Philips CM10 electron microscope. Click here for file

Additional file 10: Table S7 Gene annotation of the *C. showae* CC57C genome. Click here for file
