# Supplementary material for: Co-occurrence of anaerobic bacteria in colorectal carcinomas
Source: Microbiome. 2013 May 15;1:16. doi: 10.1186/2049-2618-1-16 (PMC3971631; doi:10.1186/2049-2618-1-16)
Supplement: Additional file 4: Table S4 — HLA allele assignments. [file 2049-2618-1-16-S4.doc]

**Table s4. CRC cohort HLA allele assignments.**

|  | **RNA-Seq predictions** | | | | | | **PCR-based HLA typing** | | | | | |
| --- | --- | --- | --- | --- | --- | --- | --- | --- | --- | --- | --- | --- |
| **Genes** | **A** | **A** | **B** | **B** | **C** | **C** | **A** | **A** | **B** | **B** | **C** | **C** |
| **Patient ID** |  |  |  |  |  |  |  |  |  |  |  |  |
| 13 | A*03 | A*24 | B*44 | B*57 | C*06 | C*16 |  |  |  |  |  |  |
| 14 | A*03 | A*24 | B*14 | B*44 | C*02 | C*08 |  |  |  |  |  |  |
| 17 | A*03 | A*23 | B*39 | B*44 | C*04 | na | A*03 | A*23 | B*39 | B*44 | C*04 | C*07 |
| 19 | A*26 | na | B*27 | B*55 | C*01 | C*03 |  |  |  |  |  |  |
| 20 | A*03 | A*24 | B*07 | B*40 | C*02 | C*07 | A*03 | A*24 | B*07 | B*40 | C*02 | C*07 |
| 22 | A*02 | A*03 | B*35 | na | C*04 | na |  |  |  |  |  |  |
| 27 | A*31 | na | B*15 B*35 B*46 B*54 B*56 | B*27 | C*01 | C*03 C*15 |  |  |  |  |  |  |
| 28 | A*02 | na | B*51 | na | C*07 | na |  |  |  |  |  |  |
| 30 | A*02 | A*24 | B*40 | B*57 | C*01 | C*07 |  |  |  |  |  |  |
| 31 | A*02 | A*24 | B*08 | B*57 | C*01 | C*03 |  |  |  |  |  |  |
| 32 | A*02 | A*11 | B*07 | B*44 | C*07 | na |  |  |  |  |  |  |
| 33 | A*01 | na | B*08 | B*56 | C*01 | C*07 |  |  |  |  |  |  |
| 34 | A*02 | na | B*15 | B*44 | C*05 | C*08 |  |  |  |  |  |  |
| 35 | A*01 | A*03 | B*08 | na | C*07 | na |  |  |  |  |  |  |
| 36 | A*02 | A*25 A*26 A*43 A*66 | B*44 | B*57 | C*05 | C*06 |  |  |  |  |  |  |
| 37 | A*02 | A*24 | B*15 B*46 | B*40 | C*01 | C*017 |  |  |  |  |  |  |
| 40 | A*02 | A*32 | B*27 | B*51 | C*01 | C*02 |  |  |  |  |  |  |
| 42 | A*01 | A*02 | B*08 | B*27 | C*01 | C*06 C*07 | A*01 | A*02 | B*08 | B*27 | C*01 | C*07 |
| 43 | A*01 A*36 | A*02 | B*44 | na | C*05 | C*07 |  |  |  |  |  |  |
| 44 | A*34 | na | B*08 | B*14 | C*05 C*08 | na |  |  |  |  |  |  |
| 47 | A*01 A*11 A*30 A*36 | A*31 | B*15 | B*40 | C*03 | na |  |  |  |  |  |  |
| 49 | A*01 | na | B*08 | B*57 | C*05 C*08 | C*07 | A*01 | na | B*08 | B*57 | C*06 | C*07 |
| 50 | A*24 | na | B*08 B*41 B*42 | B*40 | na | na |  |  |  |  |  |  |
| 52 | A*02 | A*68 | B*15 | B*44 | C*03 | C*07 |  |  |  |  |  |  |
| 53 | A*01 | A*68 | B*44 | B*51 | C*16 | na | A*01 | A*68 | B*44 | B*51 | C*16 | na |
| 54 | A*01 | na | B*08 | B*37 | C*06 | C*07 |  |  |  |  |  |  |
| 56 | A*24 | A*68 | B*15 | na | C*01 | C*03 |  |  |  |  |  |  |
| 57 | A*03 | A*24 | B*07 | B*15 | C*03 | C*07 |  |  |  |  |  |  |
| 58 | A*02 | na | B*08 | B*15 | C*03 | C*07 |  |  |  |  |  |  |
| 59 | A*01 | A*26 | B*08 | B*51 | C*01 | C*07 |  |  |  |  |  |  |
| 60 | A*02 | A*30 | B*13 | B*40 | C*03 | C*06 |  |  |  |  |  |  |
| 62 | A*01 | A*25 | B*08 | B*39 | C*07 | C*12 |  |  |  |  |  |  |
| 63 | A*01 A*36 | A*29 | B*08 | B*40 | C*03 | C*07 |  |  |  |  |  |  |
| 64 | A*03 | A*29 | B*44 | B*48 | C*08 | C*16 |  |  |  |  |  |  |
| 65 | A*02 | A*11 | B*07 | B*27 | C*02 | C*07 | A*02 | A*11 | B*07 | B*27 | C*02 | C*07 |
| 66 | A*02 | A*24 | B*35 | B*44 | C*04 | C*07 | A*02 | A*24 | B*35 | B*44 | C*04 | C*07 |
| 67 | A*02 | A*03 | B*07 | B*40 | C*03 | C*07 |  |  |  |  |  |  |
| 68 | A*02 | A*24 | B*07 | B*50 | C*06 | C*07 |  |  |  |  |  |  |
| 69 | A*02 | na | B*07 B*55 B*56 | B*27B*37 | C*01 | C*02 |  |  |  |  |  |  |
| 70 | A*01 | A*02 | B*08 | B*15 | C*04 | C*07 | A*01 | A*02 | B*08 | B*15 | C*04 | C*07 |
| 71 | A*02 | A*32 | B*44 | na | na | na |  |  |  |  |  |  |
| 73 | A*01 | A*36 | B*08 | B*57 | C*06 | C*07 |  |  |  |  |  |  |
| 74 | A*03 | A*33 | B*14 | B*51 | C*02 | C*08 |  |  |  |  |  |  |
| 75 | A*02 | A*03 | B*08 | B*39 | C*05 | C*07 | na | na | B*08 | B*39 | na | na |
| 76 | A*03 | A*11 | B*44 | na | C*02 | na |  |  |  |  |  |  |
| 79 | A*03 | A*33 | B*14 | na | C*08 | na |  |  |  |  |  |  |
| 81 | A*31 | A*68 | B*40 | B*51 | C*03 | C*15 | A*31 | A*68 | B*40 | B*51 | C*03 | C*15 |
| 83 | A*01 | A*29 | B*08 | B*44 | C*05 | C*07 | A*01 | A*29 | B*08 | B*44 | C*05 | C*07 |
| 85 | A*02 | A*24 | B*08 | B*40 | C*03 | C*07 |  |  |  |  |  |  |
| 86 | A*02 | A*03 | B*07 | B*44 | C*05 C*08 | C*07 |  |  |  |  |  |  |
| 87 | A*01 | A*11 | B*13 | B*37 | C*06 | na |  |  |  |  |  |  |
| 88 | A*01 | A*02 | B*08 | B*14 | C*03C*07 | C*08 | A*01 | A*02 | B*08 | B*14 | C*07 | C*08 |
| 89 | A*01 | A*02 | B*08 | B*14 | C*07 | C*08 |  |  |  |  |  |  |
| 90 | A*02 | na | B*40 | B*46 | C*01 | C*03 | A*02 | na | B*40 | B*46 | C*01 | C*03 |
| 91 | A*01 | A*36 | B*40 | B*55 | C*03 | C*15 |  |  |  |  |  |  |
| 92 | A*23 | A*30 | B*13 | B*44 | C*01 C*06 C*12 | C*18 |  |  |  |  |  |  |
| 93 | A*02 | A*32 | B*27 | B*51 | C*01 | C*02 |  |  |  |  |  |  |
| 94 | A*03 | na | B*13 | B*35 | C*04 | C*06 |  |  |  |  |  |  |
| 95 | A*02 | A*24 | B*18 | B*55 | C*01 | C*12 | A*02 | A*24 | B*18 | B*55 | na | na |
| 96 | A*01 | A*02 A*32 A*74 | B*07 | B*35 | C*04 | C*18 |  |  |  |  |  |  |
| 97 | A*02 | na | B*07 | B*44 | C*05 | C*07 | A*02 | na | B*07 | B*44 | C*07 | na |
| 98 | A*02 | A*31 | B*40 | B*56 | C*01 | C*03 |  |  |  |  |  |  |
| 99 | A*01 | A*03 | B*08 | na | C*03 | C*07 | A*01 | A*03 | B*08 | B*15 | C*03 | C*07 |
| 100 | A*02 | A*24 | B*40 | na | C*02 | C*03 |  |  |  |  |  |  |
| 101 | A*01 | A*02 | B*44 | B*51 | C*04 | C*15 |  |  |  |  |  |  |

na: no HLA-I predictions available from RNA-Seq or PCR-based HLA typing.

Blank: no PCR-based HLA typing attempted.

Multiple HLA-I groups (eg. C*03 C*07) indicate ambiguous RNA-Seq predictions.
