# Supplementary material for: Co-occurrence of anaerobic bacteria in colorectal carcinomas
Source: Microbiome. 2013 May 15;1:16. doi: 10.1186/2049-2618-1-16 (PMC3971631; doi:10.1186/2049-2618-1-16)
Supplement: Additional file 5: Table S5 — Differentially represented genera from top 99% microbial abundance. Sequence reads normalized for depth of sequencing were analyzed using statistical methods in the Metastats package [24], which were designed for comparing clinical metagenomic samples from two treatment populations on the basis of read count data. Rare microbes (collectively <1% of sequence data) were excluded from analysis. Genera highlighted in RED are nominally over-represented in tumor tissue relative to matched normal control tissue (P <0.05), and genera highlighted in GREEN are nominally under-represented in tumor tissue (P <0.05). Genera shown in bold indicate significantly over-represented microbes in the tumor tissue (q <0.05). [file 2049-2618-1-16-S5.doc]

**Table s5. Differentially represented genera from top 99% microbial abundance.** Sequence reads normalized for depth of sequencing were analyzed using statistical methods in the Metastats package [24], which were designed for comparing clinical metagenomic samples from two treatment populations on the basis of read count data. Rare microbes (collectively <1% of sequence data) were excluded from analysis. Genera highlighted in **RED** are nominally over-represented in tumor tissue relative to matched normal control tissue (p<0.05), and genera highlighted in **GREEN** are nominally under-represented in tumor tissue (p<0.05). Genera shown in bold indicate significantly over-represented microbes in the tumor tissue (q<0.05).

| Genus | Control  Mean  % Abundance | Tumor  Mean  % Abundance | p value | q value |
| --- | --- | --- | --- | --- |
| *Ralstonia* | 27.63 +/- 3.97 | 33.09 +/- 4.30 | 0.355 | 0.433 |
| *Bacteroides* | 25.31 +/- 2.10 | 20.13 +/- 1.80 | 0.075 | 0.222 |
| *Ruminococcus* | 8.07 +/- 0.90 | 5.37 +/- 0.73 | 0.023 | 0.135 |
| *Clostridium* | 5.44 +/- 0.70 | 4.66 +/- 0.63 | 0.411 | 0.474 |
| *Faecalibacterium* | 5.20 +/- 0.77 | 3.82 +/- 0.67 | 0.167 | 0.340 |
| ***Fusobacterium*** | **1.36 +/- 0.45** | **4.89 +/- 1.11** | **0.002** | **0.022** |
| *Alistipes* | 3.12 +/- 0.50 | 2.43 +/- 0.44 | 0.300 | 0.425 |
| *Propionibacterium* | 1.67 +/- 0.40 | 3.00 +/- 0.80 | 0.137 | 0.319 |
| *Sphingomonas* | 1.57 +/- 0.58 | 1.86 +/- 0.33 | 0.699 | 0.500 |
| *Akkermansia* | 1.75 +/- 0.56 | 1.66 +/- 0.53 | 0.911 | 0.546 |
| *Eubacterium* | 1.45 +/- 0.22 | 1.16 +/- 0.18 | 0.320 | 0.433 |
| *Renibacterium* | 0.75 +/- 0.22 | 1.40 +/- 0.73 | 0.532 | 0.483 |
| *Escherichia* | 1.19 +/- 0.35 | 0.92 +/- 0.20 | 0.559 | 0.494 |
| *Parabacteroides* | 1.31 +/- 0.20 | 0.79 +/- 0.13 | 0.026 | 0.135 |
| *Prevotella* | 0.84 +/- 0.24 | 0.93 +/- 0.29 | 0.807 | 0.512 |
| *Bifidobacterium* | 0.89 +/- 0.71 | 0.76 +/- 0.58 | 0.752 | 0.512 |
| *Dorea* | 0.95 +/- 0.12 | 0.68 +/- 0.10 | 0.096 | 0.261 |
| *Streptococcus* | 0.75 +/- 0.27 | 0.87 +/- 0.18 | 0.779 | 0.512 |
| *Odoribacter* | 0.79 +/- 0.16 | 0.73 +/- 0.20 | 0.815 | 0.512 |
| *Coprococcus* | 0.78 +/- 0.11 | 0.65 +/- 0.11 | 0.439 | 0.474 |
| *Subdoligranulum* | 0.76 +/- 0.19 | 0.64 +/- 0.18 | 0.647 | 0.497 |
| *Burkholderiales* | 0.68 +/- 0.13 | 0.53 +/- 0.10 | 0.358 | 0.433 |
| *Collinsella* | 0.68 +/- 0.15 | 0.49 +/- 0.09 | 0.242 | 0.375 |
| *Sphingopyxis* | 0.49 +/- 0.15 | 0.62 +/- 0.10 | 0.515 | 0.483 |
| *Clostridiales* | 0.46 +/- 0.06 | 0.58 +/- 0.14 | 0.463 | 0.474 |
| *Desulfovibrio* | 0.44 +/- 0.08 | 0.58 +/- 0.18 | 0.530 | 0.483 |
| *Pseudoflavonifractor* | 0.59 +/- 0.07 | 0.39 +/- 0.05 | 0.036 | 0.147 |
| *Ruminococcaceae* | 0.58 +/- 0.10 | 0.34 +/- 0.06 | 0.029 | 0.135 |
| ***Campylobacter*** | **0.05 +/- 0.01** | **0.83 +/- 0.38** | **0.001** | **0.016** |
| *Porphyromonas* | 0.42 +/- 0.14 | 0.37 +/- 0.13 | 0.795 | 0.512 |
| *Sphingobium* | 0.35 +/- 0.12 | 0.42 +/- 0.06 | 0.631 | 0.497 |
| *Eggerthella* | 0.41 +/- 0.07 | 0.33 +/- 0.06 | 0.434 | 0.474 |
| ***Leptotrichia*** | **0.02 +/- 0.01** | **0.64 +/- 0.25** | **0.001** | **0.016** |
| *Roseburia* | 0.36 +/- 0.06 | 0.28 +/- 0.07 | 0.465 | 0.474 |
| *Blautia* | 0.37 +/- 0.05 | 0.26 +/- 0.04 | 0.119 | 0.299 |
| *Pseudomonas* | 0.24 +/- 0.09 | 0.35 +/- 0.12 | 0.497 | 0.483 |
| *Novosphingobium* | 0.20 +/- 0.04 | 0.31 +/- 0.04 | 0.072 | 0.222 |
| *Enterococcus* | 0.27 +/- 0.17 | 0.22 +/- 0.16 | 0.863 | 0.532 |
| *Bradyrhizobium* | 0.21 +/- 0.06 | 0.17 +/- 0.02 | 0.626 | 0.497 |
| *Selenomonas* | 0.09 +/- 0.04 | 0.28 +/- 0.09 | 0.044 | 0.159 |
| *Haemophilus* | 0.10 +/- 0.03 | 0.25 +/- 0.13 | 0.335 | 0.433 |
| *Phascolarctobacterium* | 0.21 +/- 0.05 | 0.12 +/- 0.04 | 0.199 | 0.342 |
| *Methylobacterium* | 0.14 +/- 0.04 | 0.15 +/- 0.02 | 0.711 | 0.500 |
| *Burkholderia* | 0.13 +/- 0.04 | 0.13 +/- 0.03 | 0.919 | 0.546 |
| *Peptostreptococcus* | 0.09 +/- 0.02 | 0.15 +/- 0.04 | 0.193 | 0.342 |
| *Parvimonas* | 0.13 +/- 0.06 | 0.09 +/- 0.04 | 0.610 | 0.497 |
| *Erysipelotrichaceae* | 0.13 +/- 0.03 | 0.08 +/- 0.02 | 0.253 | 0.375 |
| *Lactobacillus* | 0.10 +/- 0.05 | 0.08 +/- 0.04 | 0.670 | 0.497 |
| *Lawsonia* | 0.08 +/- 0.01 | 0.08 +/- 0.02 | 0.720 | 0.500 |
| *Veillonella* | 0.05 +/- 0.01 | 0.09 +/- 0.05 | 0.651 | 0.497 |
| *Oribacterium* | 0.03 +/- 0.00 | 0.09 +/- 0.07 | 0.962 | 0.561 |
| *Granulicatella* | 0.04 +/- 0.02 | 0.08 +/- 0.03 | 0.192 | 0.342 |
| *Gordonibacter* | 0.07 +/- 0.03 | 0.04 +/- 0.01 | 0.212 | 0.346 |
| *Anaerotruncus* | 0.06 +/- 0.01 | 0.04 +/- 0.01 | 0.163 | 0.340 |
| *Butyrivibrio* | 0.05 +/- 0.01 | 0.04 +/- 0.01 | 0.668 | 0.497 |
| *Holdemania* | 0.06 +/- 0.02 | 0.02 +/- 0.00 | 0.007 | 0.057 |
| *Dialister* | 0.04 +/- 0.01 | 0.04 +/- 0.01 | 0.985 | 0.564 |
