# Supplementary material for: Co-occurrence of anaerobic bacteria in colorectal carcinomas
Source: Microbiome. 2013 May 15;1:16. doi: 10.1186/2049-2618-1-16 (PMC3971631; doi:10.1186/2049-2618-1-16)
Supplement: Additional file 6: Figure S1 — Microbiome profiles of normal and tumor samples do not group separately. Principal component analysis with sample types (normal, tumor) as instrumental variables, based on the abundance of 57 genera (representing 99% of the microbe abundance) in 65 normal and 65 tumor samples. Two first components were plotted using the ade4 [26] package in R and represented 68% of the variance. CRC patients (xy points) were clustered and center of gravity (with labels normal and tumor centered in each ellipse) computed for each class. [file 2049-2618-1-16-S6.doc]

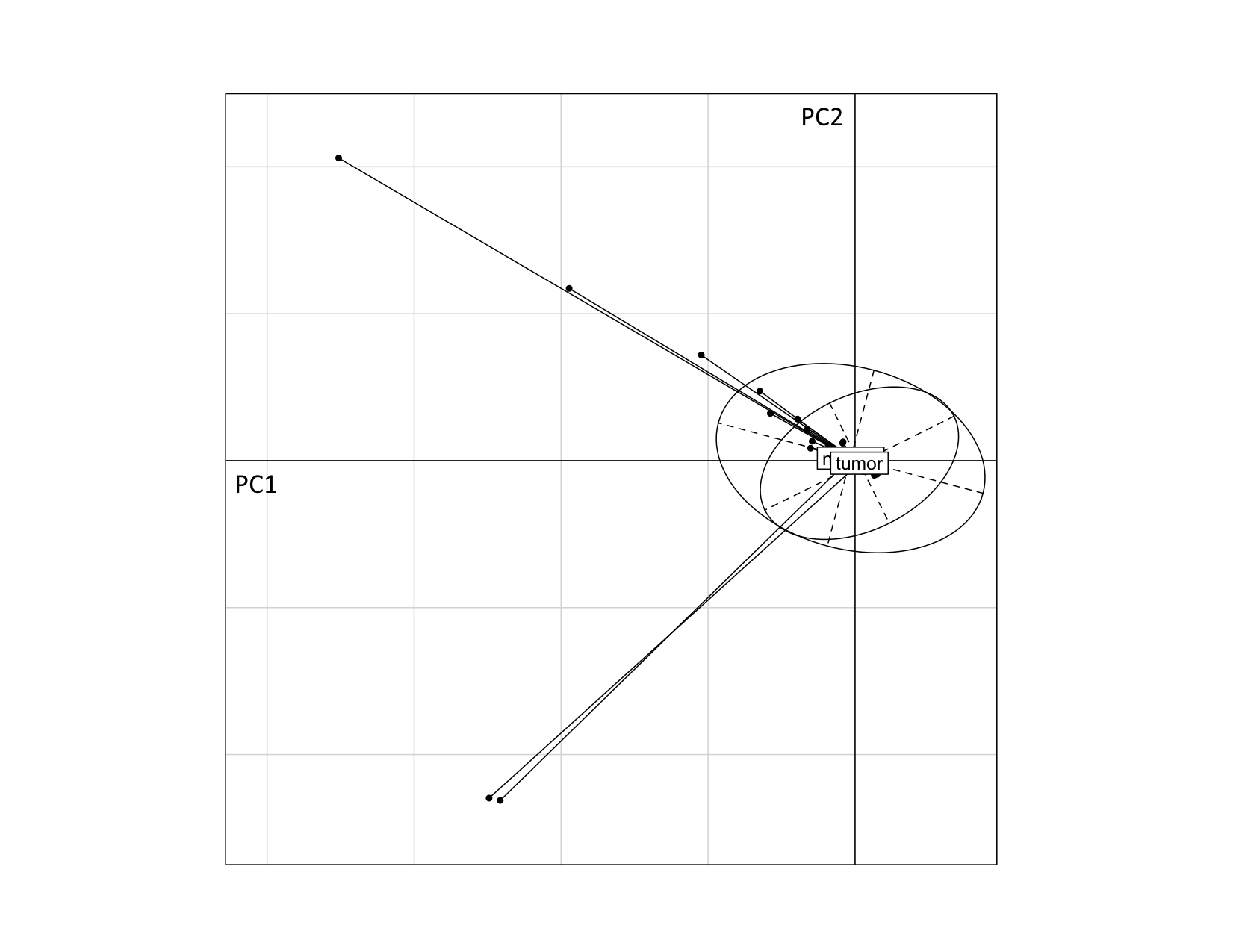


**Figure s1 - Microbiome profiles of normal and tumor samples do not group separately.** Principal component analysis with sample types (normal, tumor) as instrumental variables, based on the abundance of 57 genera (representing 99% of the microbe abundance) in 65 normal and 65 tumor samples. Two first components were plotted using the ade4 [26] package in R and represented 68% of the variance. CRC patients (xy points) were clustered and centre of gravity (with labels normal and tumor centered in each ellipse) computed for each class.
