# Supplementary material for: Co-occurrence of anaerobic bacteria in colorectal carcinomas
Source: Microbiome. 2013 May 15;1:16. doi: 10.1186/2049-2618-1-16 (PMC3971631; doi:10.1186/2049-2618-1-16)
Supplement: Additional file 7: Figure S2 — Unique read pair alignment distribution. Paired read alignments were performed as described in methods, the number of raw pairs aligning unambiguously was tallied for each transcript/sample and consolidated per Ensembl gene. [file 2049-2618-1-16-S7.doc]

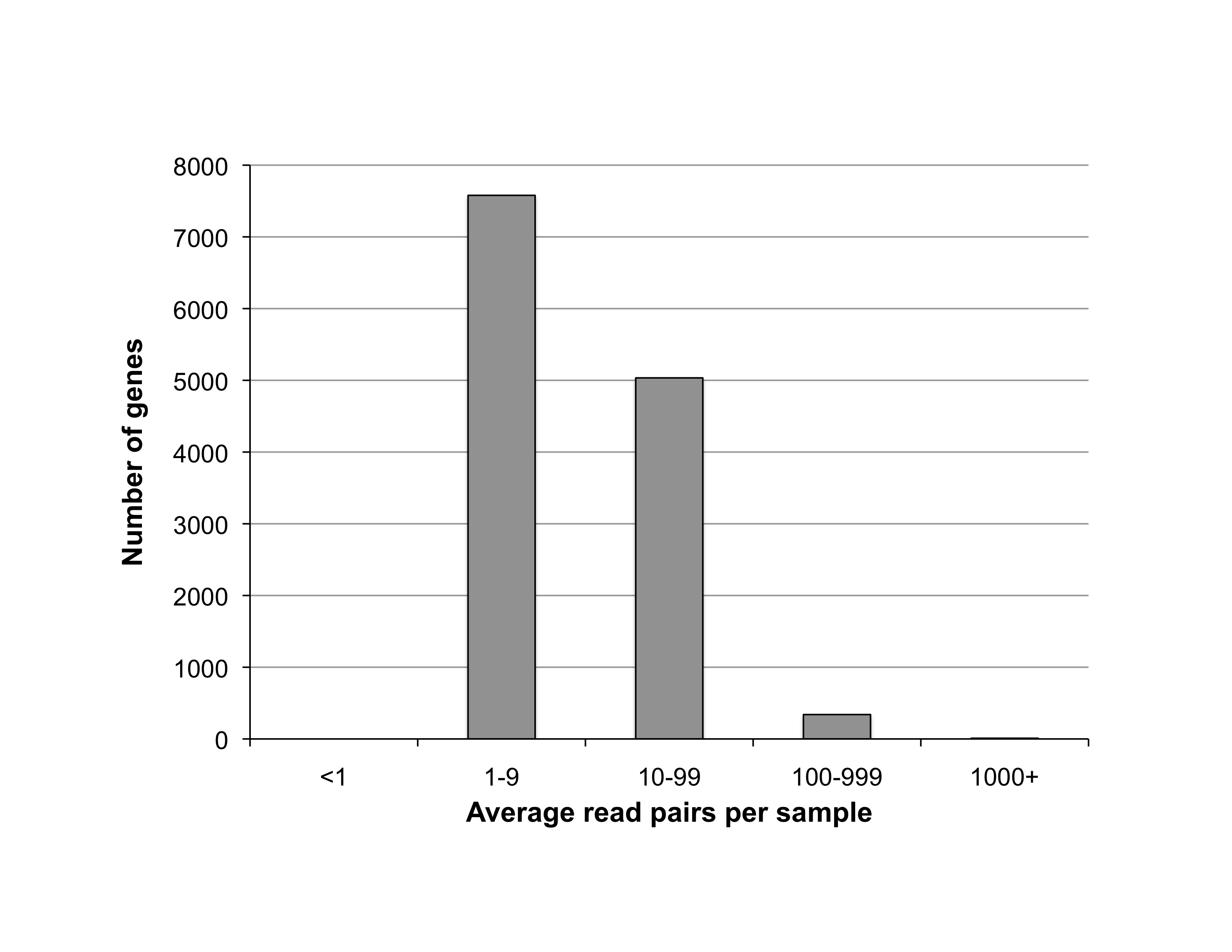


**Figure s2. Unique read pair alignment distribution.** Paired read alignments were performed as described in methods, the number of raw pairs aligning uniquely was tallied for each transcript/sample and consolidated per Ensembl gene.
