# Supplementary material for: Co-occurrence of anaerobic bacteria in colorectal carcinomas
Source: Microbiome. 2013 May 15;1:16. doi: 10.1186/2049-2618-1-16 (PMC3971631; doi:10.1186/2049-2618-1-16)
Supplement: Additional file 8: Table S6 — Microbe and host factor correlation. [file 2049-2618-1-16-S8.doc]

**Table s6**. Microbe and host factor correlation.

| Ensembl gene ID | Name | Description | Bacterial genus | Pearson R | Bootstrap p-value |
| --- | --- | --- | --- | --- | --- |
| ENSG00000197888 | UGT2B17 | UDP glucuronosyltransferase 2 family polypeptide B17 | *Ruminococcus* | 0.8414 | 0.000 |
|  |  |  | *Ruminococcaceae* | 0.7926 | 0.000 |
|  |  |  | *Pseudoflavonifractor* | 0.6212 | 0.005 |
| ENSG00000136930 | PSMB7 | proteasome (prosome macropain) subunit beta type 7 | *Campylobacter* | 0.8381 | 0.013 |
|  |  |  | *Selenomonas* | 0.8118 | 0.003 |
|  |  |  | *Fusobacterium* | 0.7152 | 0.016 |
|  |  |  | *Leptotrichia* | 0.6665 | 0.021 |
| ENSG00000183878 | UTY | ubiquitously transcribed tetratricopeptide repeat gene Y-linked | *Ruminococcus* | 0.7743 | 0.002 |
|  |  |  | *Ruminococcaceae* | 0.7432 | 0.002 |
|  |  |  | *Pseudoflavonifractor* | 0.5875 | 0.005 |
| ENSG00000101160 | CTSZ | cathepsin Z | *Campylobacter* | 0.7456 | 0.011 |
|  |  |  | *Selenomonas* | 0.7124 | 0.006 |
|  |  |  | *Fusobacterium* | 0.6156 | 0.018 |
|  |  |  | *Leptotrichia* | 0.5896 | 0.010 |
| ENSG00000169429 | IL8 | interleukin 8 | *Campylobacter* | 0.7367 | 0.000 |
|  |  |  | *Selenomonas* | 0.6803 | 0.006 |
|  |  |  | *Fusobacterium* | 0.6699 | 0.001 |
|  |  |  | *Leptotrichia* | 0.5932 | 0.010 |
| ENSG00000149196 | C11orf73 | chromosome 11 open reading frame 73 | *Campylobacter* | 0.7091 | 0.008 |
|  |  |  | *Selenomonas* | 0.6741 | 0.007 |
|  |  |  | *Fusobacterium* | 0.6184 | 0.009 |
|  |  |  | *Leptotrichia* | 0.5437 | 0.011 |
| ENSG00000067048 | DDX3Y | DEAD (Asp-Glu-Ala-Asp) box polypeptide 3 Y-linked | *Ruminococcus* | 0.6645 | 0.002 |
|  |  |  | *Ruminococcaceae* | 0.6321 | 0.003 |
|  |  |  | *Pseudoflavonifractor* | 0.5091 | 0.004 |
| ENSG00000215018 | COL28A1 | collagen type XXVIII alpha 1 | *Ruminococcus* | 0.6508 | 0.000 |
|  |  |  | *Ruminococcaceae* | 0.6402 | 0.000 |
|  |  |  | *Parabacteroides* | 0.6097 | 0.000 |
|  |  |  | *Pseudoflavonifractor* | 0.5732 | 0.002 |
| ENSG00000167244 | IGF2 | insulin-like growth factor 2 (somatomedin A) | *Campylobacter* | 0.6252 | 0.029 |
|  |  |  | *Selenomonas* | 0.5676 | 0.018 |
|  |  |  | *Fusobacterium* | 0.5290 | 0.029 |
|  |  |  | *Leptotrichia* | 0.5047 | 0.026 |
| ENSG00000012817 | KDM5D | lysine (K)-specific demethylase 5D | *Ruminococcus* | 0.6266 | 0.001 |
|  |  |  | *Ruminococcaceae* | 0.5946 | 0.002 |
| ENSG00000224608 | HLA-B | HLA class I histocompatibility antigen B-49 alpha chain | *Campylobacter* | 0.6249 | 0.008 |
|  |  |  | *Selenomonas* | 0.5869 | 0.007 |
|  |  |  | *Fusobacterium* | 0.5086 | 0.020 |
| ENSG00000196660 | SLC30A10 | solute carrier family 30 member 10 | *Holdemania* | 0.6187 | 0.001 |
|  |  |  | *Pseudoflavonifractor* | 0.6126 | 0.002 |
| ENSG00000214320 | AL139082.1 | U3 small nucleolar RNA-associated protein 14 homolog C | *Holdemania* | 0.6059 | 0.004 |
|  |  |  | *Pseudoflavonifractor* | 0.6018 | 0.023 |
| ENSG00000179674 | ARL14 | ADP-ribosylation factor-like 14 | *Ruminococcus* | 0.6051 | 0.002 |
|  |  |  | *Ruminococcaceae* | 0.5651 | 0.002 |
| ENSG00000188738 | FSIP2 | fibrous sheath interacting protein 2 | *Parabacteroides* | 0.6018 | 0.001 |
| ENSG00000175497 | DPP10 | dipeptidyl-peptidase 10 (non-functional) | *Pseudoflavonifractor* | 0.5954 | 0.007 |
|  |  |  | *Holdemania* | 0.5306 | 0.005 |
| ENSG00000125629 | INSIG2 | insulin induced gene 2 | *Holdemania* | 0.5875 | 0.002 |
| ENSG00000189227 | C15orf61 | chromosome 15 open reading frame 61 | *Pseudoflavonifractor* | 0.5793 | 0.000 |
|  |  |  | *Holdemania* | 0.5396 | 0.001 |
| ENSG00000130204 | TOMM40 | translocase of outer mitochondrial membrane 40 homolog (yeast) | *Holdemania* | 0.5760 | 0.001 |
|  |  |  | *Pseudoflavonifractor* | 0.5181 | 0.013 |
| ENSG00000114374 | USP9Y | ubiquitin specific peptidase 9 Y-linked | *Ruminococcus* | 0.5691 | 0.002 |
|  |  |  | *Ruminococcaceae* | 0.5334 | 0.002 |
| ENSG00000157212 | PAXIP1 | PAX interacting (with transcription-activation domain) protein 1 | *Holdemania* | 0.5572 | 0.000 |
| ENSG00000168591 | TMUB2 | transmembrane and ubiquitin-like domain containing 2 | *Selenomonas* | 0.5557 | 0.000 |
|  |  |  | *Campylobacter* | 0.5337 | 0.011 |
| ENSG00000184305 | FAM190A | family with sequence similarity 190 member A | *Ruminococcus* | 0.5542 | 0.003 |
|  |  |  | *Ruminococcaceae* | 0.5212 | 0.003 |
| ENSG00000244122 | UGT1A7 | UDP glucuronosyltransferase 1 family polypeptide A7 | *Pseudoflavonifractor* | 0.5455 | 0.003 |
|  |  |  | *Holdemania* | 0.5043 | 0.003 |
| ENSG00000151062 | CACNA2D4 | calcium channel voltage-dependent alpha 2/delta subunit 4 | *Holdemania* | 0.5344 | 0.001 |
| ENSG00000135698 | MPHOSPH6 | M-phase phosphoprotein 6 | *Pseudoflavonifractor* | 0.5324 | 0.006 |
| ENSG00000111726 | CMAS | cytidine monophosphate N-acetylneuraminic acid synthetase | *Holdemania* | 0.5319 | 0.004 |
| ENSG00000086548 | CEACAM6 | carcinoembryonic antigen-related cell adhesion molecule 6 (non-specific cross reacting antigen) | *Holdemania* | 0.5274 | 0.003 |
|  |  |  | *Pseudoflavonifractor* | 0.5043 | 0.009 |
| ENSG00000162604 | TM2D1 | TM2 domain containing 1 | *Holdemania* | 0.5263 | 0.001 |
| ENSG00000113083 | LOX | lysyl oxidase | *Selenomonas* | 0.5255 | 0.007 |
| ENSG00000133401 | PDZD2 | PDZ domain containing 2 | *Holdemania* | 0.5241 | 0.001 |
| ENSG00000006534 | ALDH3B1 | aldehyde dehydrogenase 3 family member B1 | *Holdemania* | 0.5101 | 0.000 |
| ENSG00000162772 | ATF3 | activating transcription factor 3 | *Pseudoflavonifractor* | 0.5069 | 0.023 |
| ENSG00000155438 | MKI67IP | MKI67 (FHA domain) interacting nucleolar phosphoprotein | *Holdemania* | 0.5036 | 0.003 |
| ENSG00000105607 | GCDH | glutaryl-CoA dehydrogenase | *Holdemania* | 0.5032 | 0.004 |
| ENSG00000132906 | CASP9 | caspase 9 apoptosis-related cysteine peptidase | *Holdemania* | 0.5024 | 0.006 |
