# Supplementary material for: Co-occurrence of anaerobic bacteria in colorectal carcinomas
Source: Microbiome. 2013 May 15;1:16. doi: 10.1186/2049-2618-1-16 (PMC3971631; doi:10.1186/2049-2618-1-16)
Supplement: Additional file 9: Figure S3 — Representative transmission electron microscopy image of Campylobacter strain CC57C, stained with 0.5% uranyl acetate. In most cases, only a single unipolar flagellum was seen associated with each cell (arrows). The lack of sharply defined edges around cells may indicate the presence of a capsule. Image taken using a Philips CM10 electron microscope. [file 2049-2618-1-16-S9.doc]

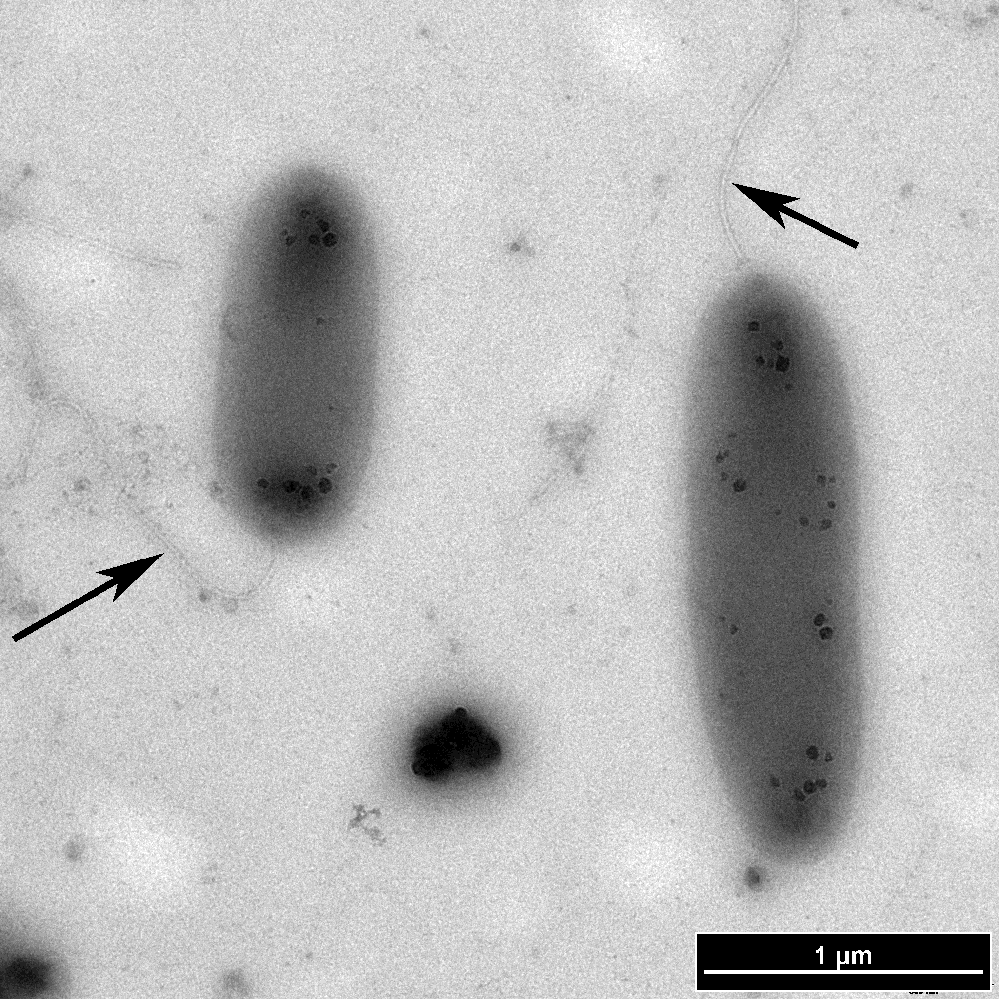


**Figure s3.** **Representative transmission electron microscopy image of *Campylobacter* strain CC57C, stained with 0.5% uranyl acetate**. In most cases, only a single unipolar flagellum was seen associated with each cell (arrows). The lack of sharply defined edges around cells may indicate the presence of a capsule. Image taken using a Philips CM10 electron microscope.
